# Vocational Rehabilitation Awareness Among Spinal Cord Injury Male Patients in Saudi Arabia: A Brief Communication

**DOI:** 10.7759/cureus.3886

**Published:** 2019-01-15

**Authors:** Ahmad H Alwashmi

**Affiliations:** 1 Physical Medicine and Rehabilitation, Qassim College of Medicine, Buriydah, SAU

**Keywords:** spinal cord injury, saudi arabia, vocational rehabilitation, awareness, employment

## Abstract

Background: Motor vehicle accidents are the most common cause of spinal cord injury (SCI) in Saudi Arabia, mainly involving young adults. Much attention has been dedicated to obtaining work after SCI during the past decades because of the psychological, social, financial, and political implications. Since high unemployment rates pose a significant social burden due to the increase in the expenditures associated with benefits, it remains an important consideration in individuals with activity limitation after spinal cord injury. There are no current data or guidelines for community reintegration or employment rates in the spinal cord injury population in Saudi Arabia.

Objective: The objective of our study was to identify the awareness of vocational rehabilitation in individuals with spinal cord injury in Saudi Arabia.

Design and methods: This cross-sectional study was conducted in the outpatient department of the largest tertiary care rehabilitation hospital in Saudi Arabia. After obtaining informed consent, structured interviews were conducted from March 2018 through July 2018 (five months) by the primary investigator and a rehabilitation nurse The interviews were administered in a one-to-one format.

Results: One hundred and twenty-one male patients with SCI were included in the study with mean age of 35.6 ± 13.9 years. Seventy (57.9) were employed at the time of injury. The employment rate decreased significantly after injury; only 20 (16.5%) were employed, 38 (31.4%) had retired, and 11 (9.1%) patients resumed their studies.

One hundred and five (86.8%) patients received rehabilitation treatment as an inpatient. Ninety-four (77.7%) reported that they had no idea about vocational rehabilitation. Only five patients (4.1%) received services of vocational rehabilitation.

Conclusion: Vocational rehabilitation awareness among spinal cord injury male patients in Saudi Arabia is lacking. This needs to be addressed to overcome unemployment and improve the quality of life which in turn may reduce the economic burden and costs among spinal cord injury patients in Saudi Arabia.

## Introduction

Traumatic spinal cord injuries (SCIs) are catastrophic to individuals and society. The motor vehicle is the main means of transportation in Saudi Arabia. Regional road traffic accidents (RTA) mortality for Saudi Arabia is high [1-2]. As a consequence, Saudi Arabia has the tragedy of having one of the highest rates of spinal cord injuries in the world [3]. Many spinal cord injury patients become discouraged to return to work because of their physical limitations [4]. Studies have shown that on the job spinal cord injury patients range from 13% to 48% [5].

In 2001, the World Health Organization (WHO) introduced The International Classification of Functioning, Disability, and Health (ICF), which describes health by including the aspect of "participation in activities" [6]. Much attention has been dedicated to obtaining work after an SCI during the past decades because of the psychological, social, financial, and political implications [7]. Among the most important consequences of being employed is the association of employment with better self-esteem, higher life satisfaction, and a sense of well-being [8]. Persons with an SCI who are not working report lower overall satisfaction and lower satisfaction with job opportunities and income [9]. This study survey was conducted to assess vocational rehabilitation awareness among spinal cord injury patients in Saudi Arabia.

## Materials and methods

This cross-sectional study was conducted in the outpatient department of the largest tertiary care rehabilitation hospital in Saudi Arabia. After obtaining informed consent, structured interviews were conducted from March 2018 through July 2018 (five months) by the primary investigator and a rehabilitation nurse The interviews were administered in a one-to-one format. Personal interviews were chosen over a collection by postal questionnaires based on research that found that there were differences in information collected when using a personal interview versus postal survey [10]. Their results detail the high potential for misclassification of occupational exposure in studies based on questionnaires, finding a tendency for over-reporting. Therefore, postal questionnaires are not considered an alternative to a job and task-specific personal interviews in epidemiological studies. The questionnaire used during the interview was prepared locally by an expert in the field of spinal cord injury rehabilitation. In addition, to be eligible for inclusion in the study, the patient needed to reside in Saudi Arabia and be discharged with persistent neurological damage but without cognitive impairment. Data were described as averages (mean ± standard deviation (SD)) and percentages (frequency %) by the Statistical Package for Social Sciences (SPSS) (IBM SPSS Statistics, Armonk, NY) and the main outcome measure was the awareness of vocational rehabilitation.

## Results

One hundred and twenty-one male patients with SCI were included in the study with a mean age of 35.6 ± 13.9 years (Table 1). Seventy-five patients (62.0%) were unmarried, and 89 (74%) were from the central and southern region of Saudi Arabia. Fifty-six patients (46.3%) were secondary school students and 33 (27.3%) were university level students (Table 2). Seven patients (5.8%) were complete tetraplegics, 16 (13.2%) were incomplete tetraplegics, 64 (52.9%) were complete paraplegics, and 34 (28.1%) were incomplete paraplegics. Seventy patients (57.9%) were employed at the time of injury; the employment rates decreased significantly after injury.

**Table 1:** Sociodemographic Data These data were collected after spinal cord injury (SCI). The majority of our patients (75 patients (62%)) were single, 60 patients (49.2%) were from the central region, and 56 patients (46.3%) were secondary school students. n: number; SD: standard deviation

Table 1					
			n (%)		
Age (yr)	mean ± SD (min, max)	35.6 ± 13.9 (17, 87)	
Marital Status	Unmarried	75 (62.0)	
	Married	46 (38.0)			
Province of residence	Northern	17 (14.1)	
	Eastern	6 (5.0)			
	Western	8 (6.7)			
	Southern	30 (25.0)			
	Central	60 (49.2)			
Education level	Illiterate or informal education	6 (5.0)	
	Primary school	12 (9.9)			
	Intermediate school	10 (8.3)			
	Secondary school	56 (46.3)			
	College or university degree	33 (27.3)			
	Higher education	4 (3.3)			

**Table 2:** History of the Injury These data were collected after the spinal cord injury (SCI) showing 70 patients (57.9%) were employed at the time of SCI. Sixteen patients (13.2%) did not have inpatient SCI rehabilitation during their admission. Ninety-four patients (77.7%) did not have any idea about vocational rehabilitation services. Only five patients (4.1%) received vocational rehabilitation during their inpatient admission. The majority of SCI patients (64 patients (52.9%)) were complete paraplegics. n: number

Table 2		
		n (%)
At the time of injury, were you employed?	No	51 (42.1)
	Yes	70 (57.9)
Have you received any intensive rehabilitation program as an inpatient?	No	16 (13.2)
	Yes	105 (86.8)
Do you have any idea about vocational rehabilitation?	No	94 (77.7)
	Yes	27 (22.3)
Have you ever received services of vocational rehabilitation?	No	116 (95.9)
	Yes	5 (4.1)
Complete tetraplegic	No	114 (94.2)
	Yes	7 (5.8)
Incomplete tetraplegic	No	105 (86.8)
	Yes	16 (13.2)
Complete paraplegic	No	61 (50.4)
	Yes	64 (52.9)
Incomplete paraplegic	No	87 (71.9)
	Yes	34 (28.1)

Only 20 patients (16.5%) remained employed, 38 patients (31.4%) retired, and 11 patients (9.1%) resumed their studies (Table 3). 

**Table 3:** Status After Spinal Cord Injury (SCI) Data showing that "unemployed and looking for a job" is the highest category post-SCI (41 patients (33.9%)), whereas only 20 patients (16.5%) were currently employed. Considering that at the time of the SCI, 70 patients (57.9%) were employed (Table 2), there was a significant drop in employment status. n: number

Table 3		n (%)
Employment status	Employed	20 (16.5)
	Unemployed and looking for a job	41 (33.9)
	Unemployed and not looking for a job	11 (9.1)
	Retired	38 (31.4)
	Student	11 (9.1)

One hundred and five patients (86.8%) received rehabilitation as an inpatient (Table 2). Ninety-four patients (77.7%) reported that they had no idea about vocational rehabilitation. Only five patients (4.1%) received the services of vocational rehabilitation.

## Discussion

In this cross-sectional study of 121 patients, 94 (77.7%) reported that they had no idea about vocational rehabilitation and only five patients (4.1%) received vocational rehabilitation services (Table 2). "Unemployed and looking for a job" was the highest category post-SCI (41 patients (33.9%), whereas only 20 patients (16.5%) were currently employed (Table 3). Considering that at the time of the SCI, 70 patients (57.9%) were employed (Table 2), there was a significant drop in employment status that needs to be investigated. Out of 121 patients, 60 patients (49.2%) were from the Central region of the Kingdom, followed by 30 patients (25%) from the Southern region, 17 patients (14.1%) from the Northern region, eight patients (6.7%) from the Western region, and six patients (5%) from the Eastern region. This difference in the numbers of patient distribution was attributed to the referral system from peripheral regions to tertiary hospitals where more proper medical and rehabilitation services were provided to the patients.   

Our study revealed that most of the spinal cord injury patients were single and young secondary school students and their injuries were mostly due to automobile crashes, which concur with the most common cause of spinal cord injury globally [11]. In fact, more than 80% of the SCI patients in Saudi Arabia are men, particularly those in the younger age group [12-16]​​​​​​. Studies also reported that traumatic spinal cord injury primarily affects males between 18 and 32 years of age, with road traffic accidents being the primary cause [17]​​​​​​​. Disability due to SCI at this young age prone these population out of the job market and the majority of these patients are not aware of vocational rehabilitation as indicated in our study. Disability due to SCI at this young age will, without doubt, contribute to this population being left out of the job market, especially if these individuals are unaware of vocational rehabilitation and its potential benefits. In fact, we found that the majority of these patients were not aware of vocational rehabilitation, at least in Saudi Arabia, as indicated in our study.

The goal of vocational rehabilitation (VR) services is to assist a person with disabilities to effectively find and sustain reasonable employment which, in turn, enhances their potential for participation in society [18]​​​​​​​. Employment for individuals with SCI represents a complex outcome that is dependent upon psychosocial characteristics, as well as the social, physical, and economic environment of any given place and time [19-20]​​​​​​​. Because of this, vocational rehabilitation is very important in assisting people in performing the behaviors necessary to move from undesirable states to more satisfactory ones [21]​​​​​​​.

Our study shows that the majority of our spinal cord injury patients did not know about vocational rehabilitation. It also highlights the importance of vocational rehabilitation awareness, and knowledge of vocational rehabilitation is crucial as this could change their quality of life and optimize their level of independence. This brief communication article is limited to one center and conducted only in male patients. Therefore, we recommend having a multicenter study that would include both genders, especially since women are currently allowed to drive in Saudi Arabia, to have a better depth of this issue.

## Conclusions

Vocational rehabilitation awareness among spinal cord injury male patients in Saudi Arabia is lacking. This needs to be addressed to overcome unemployment and improve the quality of life which, in turn, could reduce the economic burden and cost among spinal cord injury patients in Saudi Arabia.
